# Hepatocellular carcinoma: Intratumoral EpCAM-positive cancer stem cell heterogeneity identifies high-risk tumor subtype

**DOI:** 10.1186/s12885-020-07580-z

**Published:** 2020-11-23

**Authors:** Jenny Krause, Johann von Felden, Christian Casar, Thorben W. Fründt, Johanna Galaski, Constantin Schmidt, Caroline Jung, Harald Ittrich, Sören A. Weidemann, Till Krech, Asmus Heumann, Jun Li, Lutz Fischer, Guido Sauter, Ansgar W. Lohse, Henning Wege, Kornelius Schulze

**Affiliations:** 1 Department of Medicine, University Medical Centre Hamburg-Eppendorf, Hamburg, Germany; 2 Bioinformatics Core, University Medical Centre Hamburg-Eppendorf, Hamburg, Germany; 3 Department of Diagnostic and Interventional Radiology and Nuclear Medicine, University Medical Centre Hamburg-Eppendorf, Hamburg, Germany; 4 Department of Pathology, University Medical Centre Hamburg-Eppendorf, Hamburg, Germany; 5 Department of General, Visceral and Thoracic Surgery, University Medical Centre Hamburg-Eppendorf, Hamburg, Germany; 6 Department of Visceral Transplant Surgery, University Medical Centre Hamburg-Eppendorf, Hamburg, Germany; 7 Mildred Scheel Cancer Career Centre HaTriCS4, University Medical Centre Hamburg-Eppendorf, Martinistraße 52, 20246 Hamburg, Germany

**Keywords:** Hepatocellular carcinoma, Cancer stem cell features, EpCAM, Intratumoral heterogeneity

## Abstract

**Background:**

The translational interest in the intratumoral heterogeneity of hepatocellular carcinoma (HCC) has been increasing. The dismal prognosis of this pathology is linked to the features of the HCC harbouring cancer stem cells (CSC), represented by EpCAM-expression. However, the extent of the impact of intratumoral distribution of CSC-features, both on the recurrence after curative resection and on clinical outcome, remains unknown. To address this, we investigated the spatial heterogeneity of CSC-features with the aim of identifying the unique HCC patient subgroups amenable to adjuvant treatment.

**Methods:**

We designed a tissue microarray (TMA) from patients who had received liver resection between 2011 and 2017. Tumor specimens were sampled at multiple locations (*n* = 3–8). EpCAM-positivity was assessed for intensity and proportion by applying a score dividing three groups: (i) negative (E−/−); (ii) heterogeneous (E−/+); and (iii) homogeneous (E+/+). The groups were further analysed with regard to time-to-recurrence (TTR) and recurrence-free-survival (RFS).

**Results:**

We included 314 tumor spots from 69 patients (76.8% male, median age 66, liver cirrhosis/fibrosis 75.8%). The risk factors were alcohol abuse (26.2%), NASH (13.1%), HBV (15.5%), HCV (17.9%) and others (27.4%), representative of a typical Western cohort. E+/+ patients experienced significantly shorter TTR and RFS compared to E+/− and E−/− patients (TTR 5 vs. 19 months, *p* = 0.022; RFS 5 vs. 14 vs. 21 months, *p* = 0.016). Only homogeneous EpCAM-positivity correlated with higher AFP levels (> 400 ng/ml, *p* = 0.031)*.*

**Conclusions:**

Spatial heterogeneity of EpCAM-expression was markedly present in the cohort. Of note, only homogeneous EpCAM-expression correlated significantly with early recurrence, whereas heterogeneous EpCAM-expression was associated with clinical endpoints comparable to EpCAM-negativity. We identified a unique HCC subtype associated with a high risk of tumor recurrence.

## Background

Hepatocellular carcinoma (HCC) is the most frequent primary liver cancer (90%) and the 4th leading cause of cancer-related death worldwide [1]. HCC develops in 70–90% of patients with chronic liver disease, mainly those with liver cirrhosis due to chronic hepatitis B or C, alcohol abuse, non-alcoholic steatohepatitis (NASH), or rare causes such as hemochromatosis. HCC patients are a highly heterogeneous group regarding the diversity of aetiologies and presence or absence of underlying liver disease. This is displayed by intertumoral molecular and histopathological heterogeneity. There is increasing evidence underlining additional significance of intratumoral heterogeneity, as shown by *Friemel* et al., who demonstrated intratumoral diversity in 87% of HCC patients by applying morphological and immunohistochemical parameters in a small group of patients [2].

Carcinomas harbouring cancer stem cell (CSC)-features have a dismal prognosis, due to the higher level of invasiveness and greater potential in metastases-dissemination [3–9]. As a major breakthrough, *Yamashita* et al. identified EpCAM-positive cancer cell subpopulations in HCC, harbouring the potential for self-renewal, de-differentiation, tumor-initiation, invasiveness, and the capacity to form distant metastases [3, 10, 11]. Moreover, the expression of EpCAM in HCC has been linked to poor prognosis in HCC, suggesting EpCAM as a potential biomarker for risk stratification [4, 11, 12]. However, the degree to which the intensity and spatial distribution of intratumoral EpCAM-expression impacts local aggressiveness and metastases-dissemination remains unclear.

To date, the most effective therapy for HCC is resection at earlier tumor stages, however, tumor recurrence and dismal prognosis are common, because further treatment options are limited to tyrosine kinase inhibitors and palliative care. There is an urgent need to identify risk factors for early metastases-dissemination, local aggressiveness, and recurrence. These risk factors could be decisive for adjuvant treatment in these patients. So far, no adjuvant treatment has proven to be beneficial in HCC, which has been mostly related to the high degree of chemotherapy and radiation resistance of HCC [13].

Therefore, we aimed to determine the spatial heterogeneity of CSC-features by the intensity and proportion of EpCAM-positive cell-populations within different locations of HCC-nodules, and to determine its impact on recurrence and aggressive tumor behaviour. We investigated the clinical impact of intratumoral heterogeneity on outcome, discriminating three pre-defined groups: (i) EpCAM-negative (E−/−); (ii) heterogeneous (E−/+); and (iii) homogeneous (E+/+). Finally, we correlated intratumoral EpCAM heterogeneity groups with serum AFP-levels, another surrogate marker for poor prognosis [11], and major clinical endpoints such as TTR and RFS in order to understand whether EpCAM-expression pattern determines HCC subgroups with poor prognosis.

## Methods

### Patients

For this study, we screened the medical records of 987 patients with a HCC diagnosis, who had been treated at the University Medical Centre Hamburg-Eppendorf between July 2011 and October 2017. Liver resection (LR) was carried out on 69 patients and sufficient tumor tissue was available to prepare a tissue micro-array (TMA). Patients were monitored during the post-operative course and recurrence was diagnosed using cross-sectional imaging, as recommended by the guidelines of the European Association for the Study of the Liver (EASL) and the American Association for the Study of Liver Diseases (AASLD) [14, 15]. Where there was inconclusive contrast dynamics, we performed histological confirmation by biopsy. Patients under 18 years of age or who had any active or pre-existing concurrent malignancy were excluded. The study was approved by the Ethics Committee of the Hamburg Medical Association (approval number PV-3578) and all participants provided written informed consent for the study protocol prior to inclusion. Patients were not limited to any type of treatment or line of further treatment. Prior to specific TMA- and immunohistochemistry (IHC)-analysis, tissue samples were reviewed by two independent pathologists from the Institute of Pathology of the University Medical Centre Hamburg-Eppendorf.

### Clinical characteristics

Baseline characteristics such as demographic and other clinical parameters (performance status, risk factors, underlying chronic liver disease, tumor stage according to the TNM and BCLC classification), laboratory data (AFP, bilirubin, albumin, and International Normalized Ratio (INR)), and imaging were recorded prior to LR. After LR, the pathological staging was incorporated into the analysis and patients were monitored with regular contrast-enhanced cross-sectional imaging of the liver, clinical examination, and laboratory testing every 3 months in accordance with guideline recommendations [14, 15]. All patients were monitored until HCC recurrence or effective time of data analysis in July 2019.

### Tissue microarray

A TMA containing 314 tumor spots of the 69 patients was constructed. As controls, we included single tissue spots from non-cancerous liver tissue from HCC patients (*n* = 31) of the cohort described above, and liver tissue from healthy individuals (*n* = 31), and from patients suffering from liver cirrhosis without HCC (*n* = 31). Hematoxylin and eosin (H&E)-stained sections from all paraffin-embedded specimens were reviewed and tumor areas and adjacent liver tissue were marked on the slides. To generate a TMA mapping tumor heterogeneity, 0.6-mm tissue cores were punched out from 3 to 8 different locations of the index area from 1 to 3 different paraffin-embedded tissue specimens, respectively, and transferred into a TMA format as previously described by Kononen [16–18].

### Immunohistochemistry

**Fig. 1:**
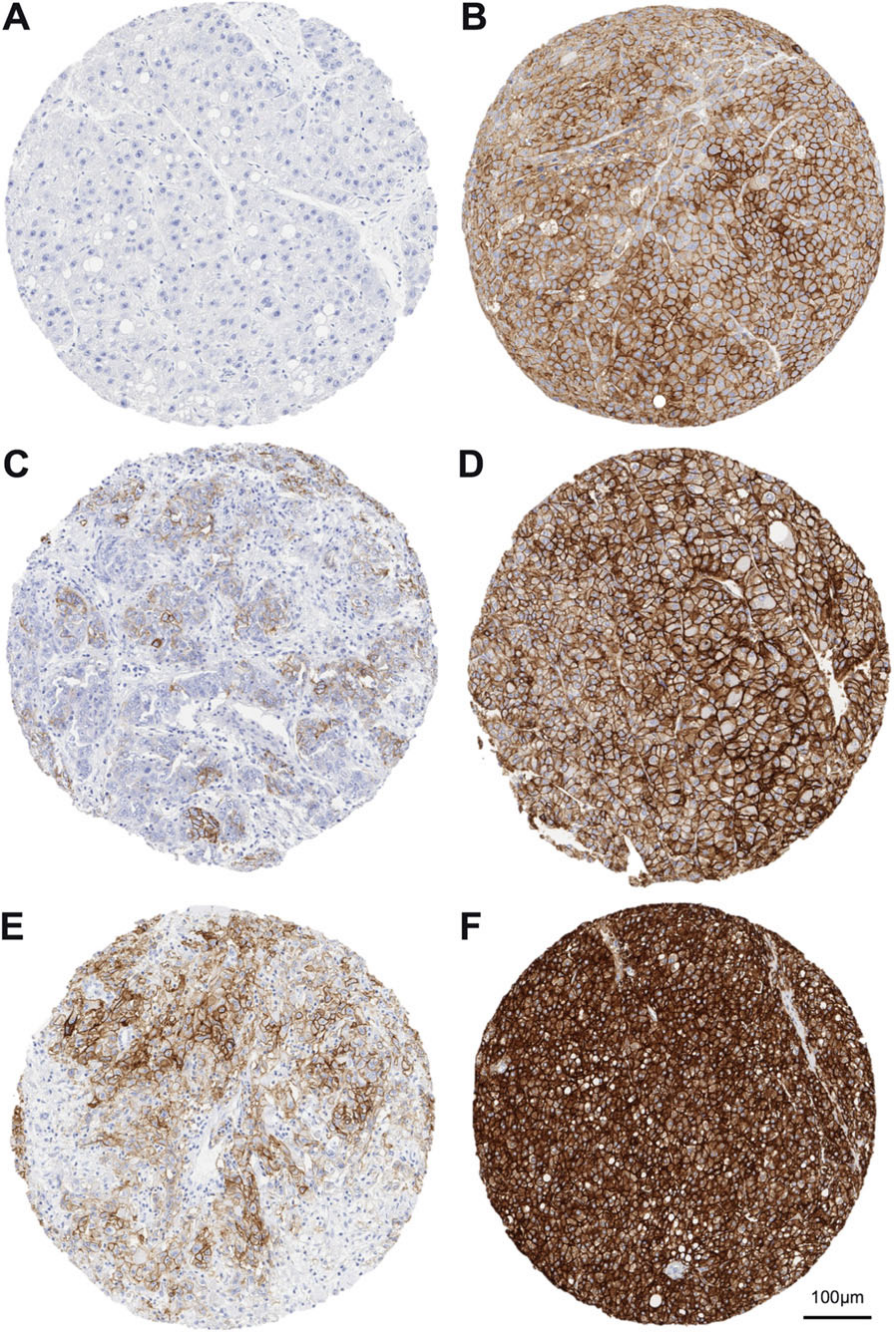
Evaluation of EpCAM Intensity and Proportion. 10fold magnification, scale bar indicating 100 μm. Proportion scoring (0 = negative, 1 < 10%, 2 = 10–50%, 3 > 50%, and 4 > 75%), intensity scoring (0 = negative, 1 = weak, 2 = moderate and 3 = strong). Proportion/Intensity: Panel **a** 0/0, **b** 4/1, **c** 2/1, **d**4/2, **e** 2/2, **f** 4/3

Freshly cut TMA sections were stained for EpCAM. High-temperature pretreatment of slides was done in a pressure cooker (DAKO buffer, pH 6.1, S1699) for 20 min. IHC was performed using a monoclonal antibody (1:10, clone VU-1D9, Novocastra, Newcastle, UK) to detect the membrane-bound positivity for EpCAM protein. The Envision system® (DAKO, Glostrup, Denmark) was used for visualization. Staining intensities and proportions of positive tumor cells were analysed for each tissue spot as proposed by the literature. A four-staged scale (0 = negative, 1 = weak, 2 = moderate and 3 = strong) was deducted for intensity, and (0 = negative, 1 < 10%, 2 = 10–50%, 3 > 50%, and 4 > 75% positivity) for proportion of positive tumor cells [19]. Fig. 1 displays representative examples of different staining results with regard to proportion and intensity of EpCAM-expression. Due to the size of the nodule, we yielded a different number of spots per tumor. Finally, heterogeneity of EpCAM-expression (intensity and proportion) was assessed by a pre-defined score, differentiating between three groups: (i) EpCAM-negative (E−/−), (ii) heterogeneous (E−/+), and (iii) homogeneous (E+/+). We considered both the proportion and intensity of EpCAM-expression within one nodule as major criteria for the classification. In detail, a heterogeneous EpCAM profile (E−/+) is defined if at least one spot is graded 0 and one spot at least 1 for intensity or proportion of EpCAM-expression.

### Measurement of serum parameters

We measured Bilirubin, Albumin and AFP within the routine clinical Dimension Vista® 1500 System by Siemens Healthineers.

The INR estimation was based on measurements of the prothrombin time by BCS® XP System by Siemens Healthineers.

### Statistical analysis

All analyses were performed using R (version 3.6, R Foundation for Statistical Computing). Univariate comparisons of clinical data between pre-defined EpCAM-classification groups were performed using Fisher’s Exact test for parametric data and Kruskal-Wallis-test for continuous data. Survival analyses and Cox-Regression modelling was performed with the survival package (version 2.38). Differences in survival rates at different time points were assessed by administrative censoring of the survival curves at the respective time point and performing a log-rank test implemented in the survdiff function.

We calculated the positive and negative prediction values, and the sensitivity and specificity of a single spot by averaging over 1000 bootstrap samplings of single spots per sample. Classification performance metrics for single spot and all spot prediction of tumor recurrence based on EpCAM intensity and proportion were calculated using the confusionMatrix function provided by the caret package (version 6.0.84). *P*-values below 0.05 were deemed statistically significant.

## Results

### Baseline parameters represent a cohort, typical of western countries

Male patients were predominant [*n* = 53 (76.8%)]. The median age was 66, ranging from 18 to 84. Of the cases, 39 (63.9%) were classified BCLC stage A. BCLC stage B [*n* = 18 (29.5%)] was less frequent, and a few cases with stage C [*n* = 4 (6.6%)] were included. The majority of patients (98.5%) were fully active or at least able to carry out work of a light or sedentary nature according to the Eastern Cooperative Oncology Group (ECOG 0 and 1). Chronic liver disease was common as underlying morbidity in our patients. Within our cohort, 41.9% of patients had liver cirrhosis (Child-Pugh A 88.5% and B 11.5%) and 24.2% of patients were treated without any histological signs of fibrosis. The remaining patients revealed fibrosis (33.9%). Known aetiologies for HCC were equally distributed across the cohort with alcohol abuse [22 (26.2%)], chronic hepatitis C [15 (17.9%)] and B [13 (15.5%)], non-alcoholic steatohepatitis (NASH) [11 (13.1%)], and rare causes or no known underlying risk factor [23 (27.4%)]. More than two thirds of the patients (73%) underwent resection for a single lesion; only a minority presented 4 or more HCC-nodules (11.1%). Pathological reviewing classified 76.8% of patients T1 or T2, 98.5% exhibited N0, 98.5% L0, and 91.3% R0. 62.3% of patients revealed neither microscopic nor macroscopic vascular invasion. The majority (71%) of tumors were moderately differentiated (G2), 15.9% well differentiated (G1), and 13% poorly differentiated (G3). Overall, our patients represented a typical cohort undergoing curative resection in Western countries. Clinical baseline and general histopathological tumor characteristics are summarized in Table 1.

**Table 1:** Clinical variables for the overall cohort. Aetiology variable may include multiples risk factors

Baseline Characteristics	Overall *n* = 69 (%)
Sex	
m	53 (76.81)
f	16 (23.19)
Age at surgical procedure (years)	
Range	18–84
Median (n)	66 (69)
ECOG (*n* = 67)	
0	37 (55.22)
1	29 (43.28)
2	1 (1.49)
BCLC (*n* = 61)	
A	39 (63.93)
B	18 (29.51)
C	4 (6.56)
Histological Stage of Liver (*n* = 62)	
normal	15 (24.19)
Fibrosis	21 (33.87)
Cirrhosis	26 (41.94)
Child-Pugh Stage (*n* = 26)	
A	23 (88.46)
B	3 (11.54)
Aetiology	
Alcohol	22 (26.19)
HBV	13 (15.48)
HCV	15 (17.86)
NASH	11 (13.1)
Other	9 (10.71)
w/o liver disease	14 (16.67)
Number of HCC-lesions (*n* = 63)	
1	46 (73.02)
2	5 (7.94)
3	5 (7.94)
> =4	7 (11.11)
T Stage (*n* = 69)	
T1	28 (40.58)
T2	25 (36.23)
T3	16 (23.19)
N Stage (*n* = 69)	
N0	68 (98.55)
N1	1 (1.45)
L Stage (*n* = 69)	
L0	68 (98.55)
L1	1 (1.45)
V Stage (*n* = 69)	
V0	43 (62.32)
V1	26 (37.68)
R Stage (*n* = 69)	
R0	63 (91.3)
R1	6 (8.7)
G Stage (*n* = 69)	
G1	11 (15.94)
G2	49 (71.01)
G3	9 (13.04)

### Spatial heterogeneity of EpCAM was markedly present

**Fig. 2:**
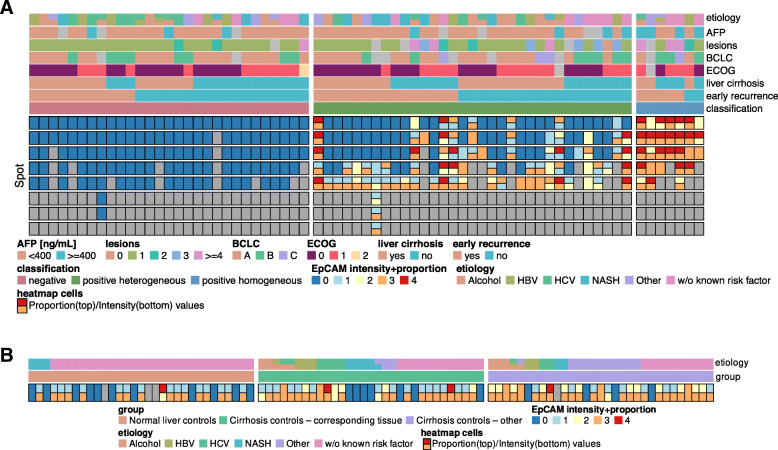
**a** Heterogeneity of EpCAM-expression in HCC. Results of EpCAM-expression per TMA spot and patient are annotated by clinical metadata. The Bottom half of the map visualises the block-wise, categorical EpCAM-Expression intensity and proportion. Map cells are split vertically to indicate different levels for intensity/proportion. **b** EpCAM-expression in control liver tissues. Results of EpCAM-expression per TMA spot and patient are annotated by clinical metadata

We confirmed the presence of spatial heterogeneity of CSC-features, measured by intensity and proportion of EpCAM-expression within the same tumor nodule. Twenty-nine patients (42%) did not exhibit any EpCAM-expression (E−/−), whereas 7 patients (10.1%) stood out with homogeneous EpCAM-expression throughout all tumor spots (E+/+). Thirty-three patients (47.8%) contained tumor spots with and without EpCAM-expression of diverse quality, confirming the presence of spatial heterogeneity of CSC-features in nearly half of patients with HCC (E−/+) (Fig. 2a). EpCAM expression of control tissues of chronic liver diseases did not correlate with aetiology or HCC recurrence (Fig. 2b). Figure 2 illustrates the results of EpCAM-staining in intensity and proportion in all 314 tumor spots with regard to EpCAM-classification score [negative (E−/−), heterogeneous (E−/+), homogeneous (E+/+)] and in correlation with baseline features.

### Only homogeneous EpCAM-expression correlated significantly with early recurrence

Based upon the EpCAM-score of the groups [(i) negative (E−/−), (ii) heterogeneous (E−/+), (iii) homogeneous (E+/+)], we were interested in the outcome of the patients in order to understand whether EpCAM-expression determined a tumor subgroup characterized by poor prognosis.

First, we checked for group comparability with regard to the baseline characteristics (Table 2). We did not find any significant differences in the demographic data, performance status, risk factors, presence or absence of underlying chronic liver disease, liver function (bilirubin, albumin, INR estimation), or staging according to the BCLC classification between the EpCAM-classification groups. Likewise, there were no significant differences between the three groups with regard to the L-, V-, N-, R- or G-stage. Overall, we did not identify any confounders between the three groups for our final outcome analysis.

**Table 2:** Cohort split into EpCAM classification groups. Group differences assessed by Fisher’s Exact test for categorical variables and Kruskal-Wallis-test for numerical variables

Baseline Characteristics	EpCAM-negative *n* = 29 (42.1%)	EpCAM- heterogeneous *n* = 33 (47.8%)	EpCAM- homogeneous *n* = 7 (10.1%)	*p*-value
Sex				
m	24 (77.42)	26 (78.79)	5 (71.43)	0.920
f	7 (22.58)	7 (21.21)	2 (28.57)	
Age at surgical procedure (years)				
range	38–84	18–81	58–76	0.326
median (n)	69 (29)	66 (33)	63 (7)	
ECOG				
0	17 (58.62)	18 (56.25)	2 (33.33)	0.535
1	11 (37.93)	14 (43.75)	4 (66.67)	
2	1 (3.45)	0 (0)	0 (0)	
BCLC				
A	19 (70.37)	18 (64.29)	2 (33.33)	0.152
B	8 (29.63)	7 (25)	3 (50)	
C	0 (0)	3 (10.71)	1 (16.67)	
Histological Stage of Liver				
No Fibrosis	6 (22.22)	6 (21.43)	3 (42.86)	0.808
Fibrosis	10 (37.04)	9 (32.14)	2 (28.57)	
Cirrhosis	11 (40.74)	13 (46.43)	2 (28.57)	
Child Pugh Stage				
A	11 (100)	11 (84.62)	1 (50)	0.111
B	0 (0)	2 (15.38)	1 (50)	
Aetiology				
Alcohol	9 (24.32)	11 (29.73)	2 (20)	0.617
HBV	7 (18.92)	4 (10.81)	2 (20)	
HCV	9 (24.32)	5 (13.51)	1 (10)	
NASH	4 (10.81)	7 (18.92)	0 (0)	
Other	4 (10.81)	3 (8.11)	2 (20)	
w/o liver disease	4 (10.81)	7 (18.92)	3 (30)	
Bilirubin				
range	0.3–1.7	0.3–2.3	0.4–0.7	0.105
median (n)	0.65 (22)	0.5 (25)	0.5 (7)	
Albumin				
range	31–43	2.34–43	30–42	0.152
median (n)	38 (20)	36 (21)	35 (6)	
INR				
range	0.93–2.42	0.92–3.08	0.95–1.25	0.863
median (n)	1.06 (25)	1.055 (24)	1.06 (6)	

**Fig. 3:**
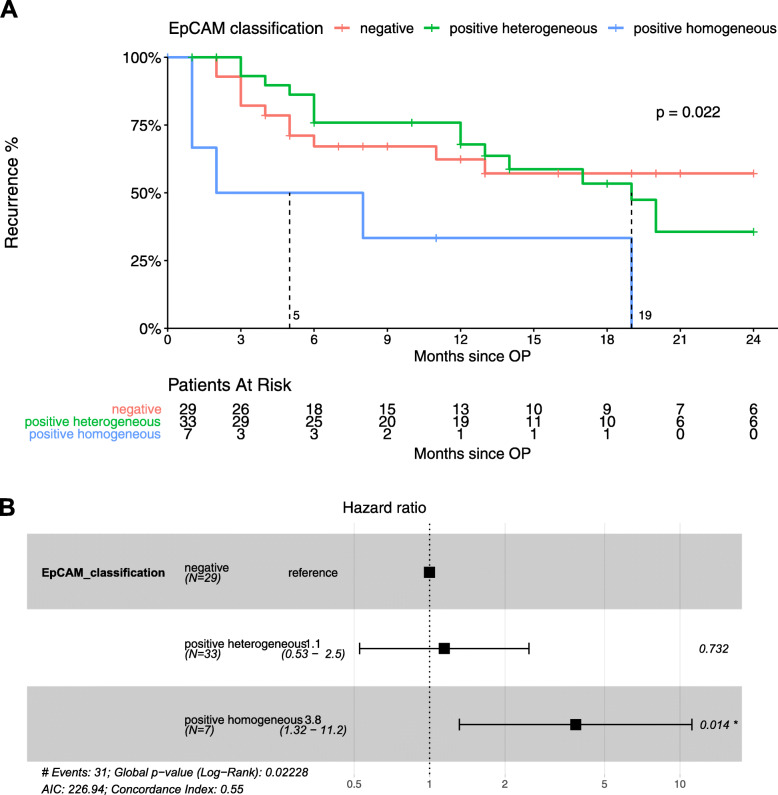
Correlation of time-to-recurrence with EpCAM classification groups. **a**: Kaplan Meier analysis showing significantly earlier recurrence within 24 months after resection of HCC with homogeneous EpCAM expression, when compared to heterogeneous EpCAM-expressing and EpCAM-negative HCC. **b.** Cox-Regression model including hazard ratios showing higher risk of recurrence within 24 months of HCC with homogeneous EpCAM expression

**Fig. 4:**
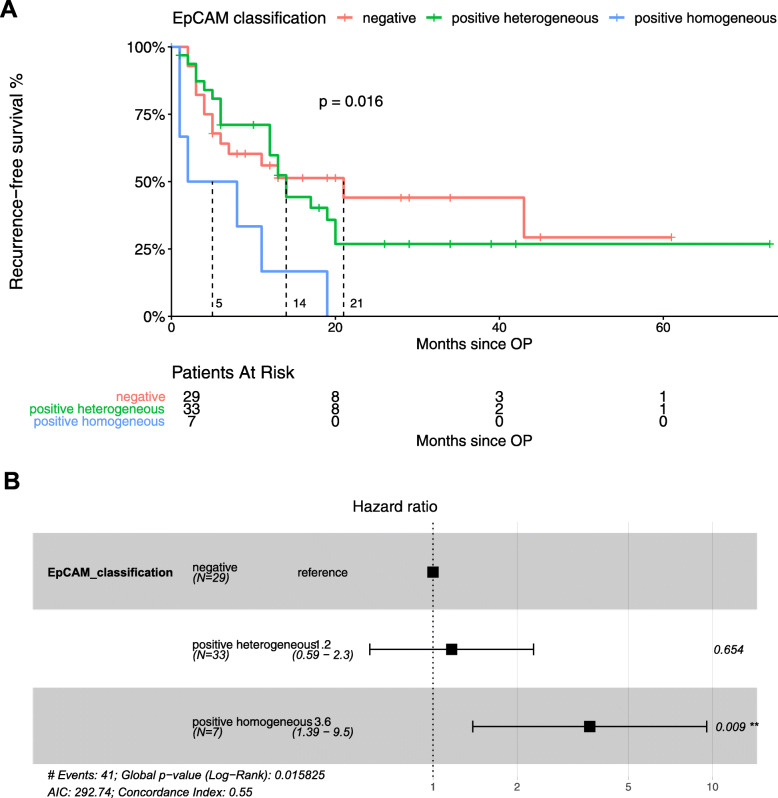
Correlation of recurrence-free survival with EpCAM classification groups. **a**: Kaplan Meier analysis showing significantly reduced recurrence-free survival of patients with HCC with homogeneous EpCAM expression, when compared to heterogeneous EpCAM-expressing and EpCAM-negative HCC. **b.** Cox-Regression model including hazard ratios showing higher risk of reduced recurrence-free survival of HCC with homogeneous EpCAM expression

To test for the clinical impact of EpCAM expression in HCC, we determined the TTR and the RFS for early recurrence (i.e. within 24 months). Recurrence within the first 24 months after curative resection is considered more likely to be true recurrence instead of de novo tumors. As highlighted in Fig. 3, TTR is significantly shorter at 5 months in patients with E+/+ compared to patients with E+/− at 19 months (*p* = 0.022). The median TTR was not reached for patients scored E−/−. The hazard ratio for recurrence between E+/+ and E−/− was 3.8 (95% CI, 1.32–11.2, *p* = 0.014). Results for RFS, depicted in Fig. 4, were also significantly different at 5 months for E+/+ compared to 14 months for E−/+, and 21 for E−/− (*p* = 0.016). The hazard ratio was 3.6 (95% CI, 1.39–9.5, *p* = 0.009). Interestingly, in both endpoints, TTR and RFS, we found a similar outcome in E+/− and E−/− groups (Figs. 3 and 4). Recurrence-free survival rates at 24 months were 57.2 and 35.6% for the E−/− and E+/− groups (p = n.s.), respectively. No patient in the E+/+ group remained without recurrence or death in the 24 months after resection. In summary, we demonstrated a significantly worse prognosis for patients with homogeneous EpCAM-expression, and also showed that heterogeneous EpCAM and negative EpCAM-expressing tumors exhibited a similar outcome, highlighting the clinical impact of thorough assessment of spatial EpCAM-expression in HCC nodules.

### Homogeneous EpCAM-expression was indicative for local aggressiveness and tumor dissemination

We observed that the group of E+/+ patients was distinct from E+/− and E−/− patients in the context of tumor aggressiveness (serum AFP-levels (</≥400 ng/mL) and number of HCC nodules). Remarkably, E+/− and E−/− demonstrated not only similar clinical behaviour, but also similar serum AFP-levels, and number of HCC nodules. So we wondered whether these surrogate parameters also statistically discriminated the group of homogeneous EpCAM-expressing HCC from the negative and heterogeneous expressing tumors, as described for EpCAM-expressing tumors in general by *Yamashita* et al. [11] Therefore, we assessed AFP serum levels prior to resection, as well as the number of satellite lesions. In HCC patients, an AFP concentration higher than 400 ng/mL has been consistently associated with poor prognosis in several treatment settings and used as the cut-off for several clinical trials such as REACH and REACH-2 [20]. AFP levels were significantly higher in the E+/+ group compared to the other groups. In detail, 57% of patients with E+/+ presented AFP levels > 400 ng/mL compared to 13.8% in E+/− and 11.5% in E−/−) (*p* = 0.031). Correspondingly, patients with E−/− and E+/− status harboured significantly less satellite tumor lesions at the time of resection compared to the E+/+ group (*p* = 0.006) (Table 3.)

**Table 3:** Surrogate markers for local aggressiveness, de-differentiation, tumor-initiation, and invasiveness in patients with HCC, considering EpCAM classification groups. Group differences assessed by Fisher’s Exact test for categorical variables and Kruskal-Wallis-test for numerical variables

Surrogate marker for CSC-features	EpCAM-negative *n* = 29 (42.1%)	EpCAM- heterogeneous *n* = 33 (47.8%)	EpCAM- homogeneous *n* = 7 (10.1%)	*p*-value
Number of HCC nodules				
1	25 (89.29)	19 (65.52)	2 (33.33)	***0.006***
2	2 (7.14)	2 (6.9)	1 (16.67)	
3	0 (0)	5 (17.24)	0 (0)	
≥ 4	1 (3.57)	3 (10.34)	3 (50)	
Serum AFP-levels				
< 400	23 (88.46)	25 (86.21)	3 (42.86)	***0.031***
≥ 400	3 (11.54)	4 (13.79)	4 (57.14)	

In summary, homogeneous EpCAM-expression was significantly indicative for higher local aggressiveness and earlier tumor dissemination compared to heterogeneous EpCAM-expression. EpCAM-negative tumor nodules demonstrated similar low aggressiveness in tumor features like EpCAM-heterogeneous tumor nodules.

### The positive predictive value of a single biopsy to diagnose HCC with homogeneous EpCAM-expression was only 45%

Since only homogeneous EpCAM-expression was associated with more aggressive tumor behaviour and subsequently, a poorer outcome, we were interested in the predictive value of a single biopsy towards the overall EpCAM-score (E+/+, E+/−, or E−/−). Therefore, we assessed the positive and negative prediction values, and the sensitivity and specificity of the EpCAM expression pattern of a single spot. We discovered that the sensitivity for a single spot was 100%, the specificity was 87%, the negative predictive value was 100%, but the positive predictive value was only 45%. Moreover, the positive predictive value of a single spot for early recurrence within 24 months after resection was 58%, the negative predictive value was 58.5%, the sensitivity was 27%, and the specificity was 84%.

## Discussion

Personalized medicine and clinical decision-making have been seeing an increase in the phenotypic and histopathological characterization of malignancies. In HCC, however, adjuvant treatment in patients has not yet been established because of the lack of positive phase 3 clinical trials, likely due to insufficient patient selection in recent studies. It is believed that certain patients could benefit from approved treatment modalities, e.g. transarterial chemoembolization (TACE), tyrosine kinase inhibitors, and immune checkpoint inhibitors, in an adjuvant setting under consideration of stringent selection criteria. Similar to colorectal cancer, high or low risk features for early metastases-dissemination and local aggressiveness could be key for adjuvant treatment after curative-intended resection. For this reason, CSC-features, in particular EpCAM-expression, are a highly promising surrogate marker in HCC identification of patient groups at risk. In addition, stratification on the basis of the underlying tumor subtype is urgently required in HCC patients. However, the extent to which intratumoral heterogeneity of EpCAM expression is present in HCC nodules, and how it might affect clinical outcome of patients has never before been evaluated.

To our knowledge, our study is the most extensive one of its kind to investigate the spatial CSC heterogeneity in HCC. We used it to verify that EpCAM-expression was heterogeneously distributed within tumor nodules. Homogeneous EpCAM-expression was present in only 1 out of 10 patients, whereas EpCAM heterogeneity was found in every other patient. EpCAM-expression of chronic liver disease control tissues did not correlate with aetiology or HCC recurrence (Fig. 2b). Homogeneous distribution of EpCAM-expression led to earlier dissemination, recurrence and/or death after curative-intended resection when compared with heterogeneous expressing tumors.

When assessing clinical baseline characteristics, we did not observe any differences in risk factors between the groups. Importantly, group determination was not influenced by the tumor status at the time of resection (BCLC), by the parameters of liver function such as bilirubin, albumin and INR estimation, or by the aetiology of the underlying liver disease. However, homogeneous distribution of EpCAM-expression was significantly associated with higher serum AFP-levels (*p* = 0.03) and the number of intrahepatic satellite HCC lesions (*p* = 0.006) compared with heterogeneous EpCAM expression, confirming former studies of Bae et al. and Yamashita et al. [11, 21] Of note, all E+/− and E−/− cases had similar clinical outcomes, suggesting the same biological behaviour in HCC with none or low expression pattern of CSC features. In short, our findings identified a new subgroup of HCC patients at high risk of early recurrence and death.

Hepatic stem and progenitor cells can be the source of tumor initiation and CSC can drive hepatocarcinogenesis [22, 23]. Moreover, EpCAM expression is linked to CSC features in various forms of cancer such as gastric, prostate, pancreatic and breast. What’s more, it is linked to a dismal prognosis and to chemotherapy resistance [6–9]. Our study highlights that only a full-scale CSC milieu within HCC nodules was of clinical significance in terms of prognosis, affirming and specifying previous findings [4, 11, 24].

Substantial EpCAM-expression is known to be the result of a distinct underlying molecular and mutational profile as introduced by molecular classifications [25]. Further studies investigating the transcriptional characterization of EpCAM-expressing tumor cells are required. HCC can be categorized into proliferation and non-proliferation classes. EpCAM positivity and abundant CSC features are associated with the G1/S2/iCluster 1, termed the progenitor group. This group is characterized by *RPS6KA3*, *TP53*, and *AXIN1* mutations and by IGF1R, AKT/mTOR signalling [25]. Thus, it will be crucial to identify the underlying mutational profile, eventually leading to HCC with homogeneous or heterogeneous EpCAM distribution. WNT-ß-catenin activation via transcription factor TCF-4 is also associated with transcriptional activation of EpCAM expression [26]. A Japanese study demonstrated in two human HCC samples inactivating *TP53* mutations (c.844C > T; c.767C > T) in EpCAM positive HCC [23]. In HCC, activating *CTNNB1* mutations and inactivating *TP53* mutations are major oncogenic events with frequencies of up to 37 and 24%, respectively [27, 28]. They could in part be reasonable drivers in homogeneous distribution in EpCAM expression. Due to methodological limitations of the present study, analysis of the mutational background was not within the scope of this work, but future studies will need to address the molecular and genetic impact on homogeneous or heterogeneous CSC patterns. Ultimately, it will be necessary to study CSC heterogeneity within a larger patient cohort at a clonal level through single cell analysis. The impact will need to be confirmed by functional experiments. In a proof of concept study, Ho et al. performed scRNA-sequencing on dissociated HCC tumor cells, and observed two subpopulations distinguished mainly by the expression of EpCAM and stemness-related genes, underlining the significance of this finding, even though the study was limited to a single specimen [29].

Our comprehensive data allowed us to demonstrate that clinical parameters, mainly risk factors and presence of underlying chronic liver disease, are not involved in determining the fate of CSC expression pattern in HCC.

We believe that histopathological investigation of resected HCC lesions is key to stratify patient management. With regard to intensity and proportion of CSC features, a simple EpCAM score could estimate the risk of recurrence. Subsequently, the score could be used to establish a clinical trial to investigate potential adjuvant treatment options where patients could be stratified in groups using the score proposed in this study.

Several trials in HCC, predominantly focusing on combinatory treatments with checkpoint inhibition, are currently underway. It is well known that PD-1 or PD-L1 expression within the tumor lesion and microenvironment in general, are not sufficient surrogate markers for treatment response in patients receiving checkpoint inhibition in HCC [25]. Recently, several study groups have emphasized that mesenchymal stem cells suppress inflammation and block the powerful adaptive immune response by initiating co-inhibitory receptor expression like PD-1/PD-L1 [30, 31]. To our knowledge, there is only minor clinical evidence in large HCC cohorts to support the efficacy of immune checkpoint inhibitors in CSC-predominant HCC subtypes. It could be elucidating to investigate the PD-1 and PD-L1 status in HCC nodules harbouring homogeneous CSC-distribution and its response to checkpoint inhibitors. Eventually, patients under mono-therapy of immune checkpoint inhibition need to be co-tested for EpCAM, PD-1, and PD-L1 expression in order to obtain the predictive value towards treatment response.

Our study specifically highlighted the impact of intratumoral heterogeneity in HCC. In other gastrointestinal forms of cancer, intratumoral heterogeneity plays a major role in clinical decision-making, e.g. HER2/neu expression in gastric cancer [32]. In the study of Warneke et al., sampling procedures posed a significant risk of generating a false-negative Her2/neu status. 25% of patients with gastric cancer and Her2/neu overexpression have been neglected due to sampling errors, leading to withholding of effective treatment with trastuzumab. Additionally, a minor false-positive rate of 6% has been reported [32].

The indication to perform biopsies in HCC patients in late disease stage will increase in the future when patient distribution is required for new treatment modalities. For this reason, we addressed the issue of representative sampling, taking into account the EpCAM expression patterns of a single spot compared with all spots of one HCC nodule and its significance towards the EpCAM score. We found that every other patient with a positive EpCAM result from biopsy actually harbours heterogeneous EpCAM-expression, and therefore has the same prognosis of a patient with EpCAM negative HCC. In conclusion, one single biopsy does not seem to be sufficient to predict patient outcome based on EpCAM-expression. However, our results were generated from patients in early tumor stages. Hence, our conclusions cannot be transferred to stratify patients with advanced tumor stages. However, we do believe the issue of sampling errors needs to be addressed in patients in later stages, because cancers with CSC features are more resistant to systemic chemotherapy [13]. The results of the present study and its impact on clinical outcome suggest that more than one biopsy is required in order to minimize the risk of false negative or positive predictive histopathological staining.

## Conclusion

CSC intratumoral heterogeneity is present in HCC nodules as demonstrated in this extensive cohort. Of note, we were able to identify one distinct HCC subgroup, which was associated with higher tumor aggressiveness, metastases-initiation and worse clinical outcome, indicated by shorter TTR and RFS. Of note, it was characterized by homogeneous distribution of EpCAM-expression. HCC with heterogeneous EpCAM-expression had characteristics remarkably similar to HCC without any EpCAM-positive tumor cells. Especially in times when significance of tumor biopsies in late stage disease is increasing, our study highlights the risk posed by sampling errors in clinical decision-making.

## Data Availability

The datasets analysed during the current study are available from the corresponding author on reasonable request.

## Abbreviations

AASLD: American Association for the Study of Liver Diseases
AFP: Alpha-Fetoprotein
AKT/mTOR: Protein Kinase B/mechanistic Target of Rapamycin
*AXIN1*: Axis Inhibition Protein 1
BCLC: Barcelona Clinic Liver Cancer Classification
CSC: Cancer Stem Cell
*CTNNB1*: Catenin Beta 1
EASL: European Association for the Study of the Liver
ECOG: Eastern Cooperative Oncology Group
EpCAM: Epithelial cell adhesion molecule
H&E: Hematoxylin and Eosin
HBV: Hepatitis B Virus
HCC: Hepatocellular Carcinoma
HCV: Hepatitis C Virus
HER2/neu: Human Epidermal Growth Factor Receptor 2
IGF1R: Insulin-Like Growth Factor 1
IHC: Immunohistochemistry
INR: International Normalized Ratio
LR: Liver resection
NASH: Non-alcoholic Steatohepatitis
PD-1: Programmed Death-1
PD-L1: Programmed Death Ligand-1
RFS: Recurrence-Free-Survival
*RPS6KA3*: Ribosomal Protein S6 Kinase A3
TACE: Transarterial Chemoembolization
TCF-4: Transcription factor 4
TMA: Tissue Microarray
TNM: TNM Classification of Malignant Tumors (T, tumor; N, lymph nodes; M, metastases)
*TP53*: Tumor suppressor protein 53
TTR: Time-To-Recurrence
WNT: Wingless-Type MMTV Integration Site Family
